# Development of a Recommendation Engine to University Student Mental Health Support Aligned With Stepped Care: Longitudinal Cohort Study

**DOI:** 10.2196/72669

**Published:** 2025-09-17

**Authors:** Pedro Velmovitsky, Charles Keown-Stoneman, Kaylen J Pfisterer, Julia Hews-Girard, Joseph Saliba, Shumit Saha, Scott Patten, Nathan King, Anne Duffy, Quynh Pham

**Affiliations:** 1 Centre for Digital Therapeutics Toronto General Hospital Research Institute University Health Network Toronto, ON Canada; 2 Institute of Health Policy, Management, and Evaluation Dalla Lana School of Public Health University of Toronto Toronto, ON Canada; 3 Dalla Lana School of Public Health University of Toronto Toronto Canada; 4 Applied Health Research Centre Li Ka Shing Knowledge Institute St Michael's Hospital Toronto Canada; 5 Systems Design Engineering University of Waterloo Waterloo Canada; 6 The Mathison Centre for Mental Health Research & Education Cumming School of Medicine University of Calgary Calgary, AB Canada; 7 Faculty of Nursing University of Calgary Calgary Canada; 8 Alberta Children's Hospital Research Institute Alberta Children's Hospital Calgary Canada; 9 Department of Biomedical Data Science School of Applied Computational Sciences Meharry Medical College Nashville United States; 10 Department of Psychiatry Queen's University Kingston, ON Canada; 11 Department of Public Health Sciences Queen's University Kingston, ON Canada; 12 Department of Psychiatry University of Oxford Oxford United Kingdom; 13 Telfer School of Management University of Ottawa Ottawa Canada; 14 School of Public Health Sciences Faculty of Health University of Waterloo Waterloo Canada

**Keywords:** mental health, university students, machine learning, depression, anxiety, stress, university, stepped care, prevention, early intervention, early detection

## Abstract

**Background:**

Mental health challenges are prevalent among Canadian higher education students, with significant rates of depression and anxiety often going untreated due to reduced early detection, stigmatizing beliefs, and practical barriers. The U-Flourish longitudinal electronic survey study launched in 2018 engages new cohorts of incoming undergraduate students and repeatedly collects data about mental health and well-being and access to support.

**Objective:**

U-Flourish survey data provide a unique opportunity to train evidence-based prediction risk models and a personalized recommendation engine to signpost students to indicated mental health support based on their own data.

**Methods:**

Two approaches were integrated in developing the risk prediction models and recommendation engine: (1) clinically defined rules by experts in the field to detect current and predict the risk of future anxiety and depression and to signpost students to appropriate care using a stepped care approach and based on clinical factors (ie, self-harm and suicidal thoughts, symptom levels, and lifetime history); and (2) machine learning models, trained with additional data including family history, early adversity, and stress indicators, to predict future risks of clinically significant depression (9-item Patient Health Questionnaire) and anxiety (7-item Generalized Anxiety Disorder questionnaire). Models were created using the XGBoost algorithm and a 70:30 ratio for training and testing with 10-fold cross-validation.

**Results:**

In total, 27.5% of students at entry to university from 2018 to 2023 were identified as having potentially clinically significant levels of anxiety and depression and signposted to university mental health services based on the clinically defined rules. Optimizing thresholds to reduce false negatives, the machine learning models predicted anxiety and depression over the year in students screening negative at baseline with accuracy comparable with reported clinical screening as evidenced by sensitivity ≥90% for all models trained. Models had high negative predictive value (≥89%), balanced against low specificity. Individuals identified at risk for anxiety or depression were signposted primarily to self-guided resources supporting proactive prevention. Model findings also demonstrated that abbreviated screens (2-item Patient Health Questionnaire [PHQ-2] and 2-item Generalized Anxiety Disorder Questionnaire [GAD-2]), with potential to reduce respondent burden and improve adherence, can be used without compromising sensitivity. Indeed, PHQ-2 displayed a 90% sensitivity and GAD-2 displayed a 92% sensitivity. Shapley additive explanations analyses revealed other predictive factors including childhood trauma, family history of mental illness, and functional impairment associated with reported depression and anxiety symptoms.

**Conclusions:**

The risk prediction models and recommendation engine’s dual approach rationalize support allocation and promote targeted early intervention and prevention, potentially improving capacity to address the increasing burden on university mental health services. Future directions include further refinement based on a larger harmonized and enriched dataset, independent validation, and implementation studies to estimate the complex factors that influence uptake, reach to services, and acceptability across more diverse student users.

## Introduction

Approximately one third of Canadian postsecondary students screen positive for clinically significant symptoms of depression or anxiety [1]. Young adults transitioning to university are particularly vulnerable. They are faced with increased responsibility for managing their daily lives including the demands of higher education, greater autonomy, and making new relationships while becoming more independent from previous support systems [1]. Furthermore, the transition to university coincides with the peak period of risk for the emergence of mental disorders—with anxiety and depression being the most common [1]. Untreated, mental health disorders are associated with a number of negative outcomes likely to affect economic stability (eg, reduced employment opportunities), education access and quality (eg, poor academic performance and increased risk of school dropout), and quality of life (eg, persisting and increasing mental disorders) [2-4]. However, less than 10% of students across Canadian university campuses receive treatment, often due to stigma associated with mental health care, not understanding how or when to reach out for support, and other practical barriers such as perceived lack of time [5-7]. Despite the low proportion of symptomatic students seeking care, student demand for mental health services is outpacing service capacity. For example, between 2009 and 2015, counseling service utilization rates at universities were 8 times greater than enrollment growth [8]. This also reflects that university counseling is the most frequently visited campus mental health service for students with a spectrum of needs and severity of concerns [9,10].

The U-Flourish Study, launched at Queen’s University in 2018, is a longitudinal successive cohort study of undergraduates designed to understand mental health trajectories and associated academic outcomes among university students. The study collects biannual data (at entry and completion of each academic year) from incoming and continuing undergraduates, including mental health symptoms, risk and lifestyle factors, and help seeking through validated measures. Examples of collected metrics include the 9-item Patient Health Questionnaire (PHQ-9) [11,12] and the 7-item Generalized Anxiety Disorder questionnaire (GAD-7) [13,14]. Identifiers are removed from the dataset. Early findings confirm high rates of anxiety and depressive symptoms among incoming students, which are associated with distress, academic impairment, and lifestyle challenges, including sleep difficulties and substance use. Data collection continued through the COVID-19 pandemic which, as anticipated, led to a worsening of mental health issues—students’ sleep and mental health quality were significantly negatively affected, with increasing rates of insomnia, anxiety, and depressive symptoms as pandemic restrictions intensified [15-17]. Self-harm and suicidal ideation also worsened, particularly among female students [17]. On one hand, as confirmed by the U-Flourish study, mental health conditions are increasingly prevalent among students, who often fail to seek treatment; on the other hand, help-seeking students are already overburdening university resources. This is an unsustainable situation and has been recognized as a mental health crisis [18].

In this context, there is a gap that needs to be addressed, namely, early detection and mapping of at-risk students to individually indicated support. This can be done by leveraging student data to proactively assess their mental health status and provide actionable feedback while reducing the burden on health care services. In other words, there is a need for innovative and rationalized models of care that can meet individual student needs while efficiently using existing campus resources. Student health services are under pressure to facilitate access to students with unmet needs, while ensuring that scarce resources are used with maximum efficiency.

The primary aim of this work was to develop and evaluate risk prediction models and an engine that combined 2 different approaches to provide treatment recommendations and signpost (ie, recommend) students to indicated levels of support rationalized in accordance with a stepped care model and based on student mental health data using (1) clinically defined rules and (2) machine learning (ML)–based predictive models. Specifically, we sought to explore the use of predictive factors such as mental health symptoms, substance use, and lifetime mental health history in a novel recommendation engine, which uses clinically defined rules and predictive models to signpost students to appropriate stepped levels of care. Such a digital resource would provide personalized recommendations in nonstigmatizing language and be available upon demand, increasing efficiency in support delivery, and potentially improving student mental health outcomes. If successful, this system and the general approach described in this paper could be implemented and scaled at other universities and higher education institutions.

The secondary aims of this work were to (1) inform future implementation of the recommendation engine into service delivery through exploration of alternative metrics (ie, the abbreviated questionnaires 2-item Patient Health Questionnaire [PHQ-2] and 2-item Generalized Anxiety Disorder Questionnaire [GAD-2] to reduce respondent burden) and (2) determine the strongest risk predictors of clinically significant anxiety or depression over the academic year, which could be used to further personalize prevention recommendations to mitigate future risk.

While many digital mental health interventions have been proposed to help students navigate university resources and provide support (eg, scheduling of appointments and self-guided resources) [19-21], these services are typically a “one-size-fits-all” solution to students and, as such, do not follow a stepped care approach, essential to provide appropriate personalized support recommendations to students and rationalize and reduce the burden on health care services. On the same token, such solutions are usually directed toward specific universities rather than providing a general approach that can be adapted and customized for different institutions. Finally, digital solutions for students are typically not co-designed with clinicians and do not apply more advanced analytical methods, such as ML, to gain further insights into student mental health. This work will address these aspects by co-designing the solution with experts, applying a hybrid rule-based and ML-based approach, and providing a general stepped care framework that can be adapted by different institutions.

## Methods

### Study Design

This work involved a systematic and phased approach to the design, development, and evaluation of risk prediction models, and a mental health recommendation engine that leveraged the richness of student data available from the longitudinal prospective U-Flourish study.

Requirements were defined by the team as follows. (1) Users were undergraduate university students. (2) The recommendation engine was not meant to replace in-person assessment or clinical triaging but rather to provide useful feedback to students about their estimated current mental health and risk over the academic year and automated recommendations only. (3) Prediction models and recommendation engine inputs were to be student-reported, mental health and mental health-related psychosocial and lifestyle variables comprising a subset of the U-Flourish survey self-report data. (4) These data were to be evidence-based and expert-informed as having strong predictive validity with mental health outcomes. (5) Recommendation outputs were to signpost students to the least intensive care level appropriate for their reported mental health status, that is, they were to follow a stepped care approach.

Next, we first describe the U-Flourish study data.

### U-Flourish Data

The U-Flourish Student Well-being Survey study [22] was launched at Queen’s University in the Fall of 2018. After a robust student-led engagement campaign, all incoming first-year students were invited to complete a web-based well-being survey via their university email. Students who completed the baseline Fall survey in mid-September were then invited to complete a Spring follow-up survey at the end of the academic year (mid-March) before final examinations. Fall and Spring surveys were subsequently sent out to students who completed each prior survey for up to 5 years. This study used data from the first 5 cohorts of students who participated in the U-Flourish survey, spanning the 2018-2019 to 2022-2023 academic years, and who had completed a follow-up survey in the subsequent Spring term. U-Flourish includes responses from 10,823 students with up to 5 years of follow-up data; a total of 4843 (44.8%) students completed at least 1 follow-up survey. This dataset is used to inform this work.

The baseline U-Flourish survey collected information on demographics, distal and proximal risk factors for mental health problems, and measures of current mental health and well-being using validated measures [22]. Subsequent follow-up surveys collected repeated data on psychosocial risk factors, mental health and well-being, and experiences over the academic year, including with mental health services. The specific subset of variables used in this study, defined in conjunction with the study clinicians, includes recreational drug use and binge drinking (Table 1), symptoms of depression (PHQ-9) [23], anxiety (GAD-7) [24], personal and familial history of mental illness, adverse childhood experiences, suicidal thoughts and behaviors and self-harm (Columbia-Suicide Severity Rating Scale, C-SSRS [25]), and self-perceived stress (Perceived Stress Scale–4 [PSS-4]) (Table 2).

Each survey was completed in a 2-week window with email reminders sent out at regular intervals. The survey was run on the Qualtrics platform. Participation was encouraged through a student-led engagement campaign that targeted first-year students and included in-class presentations, web-based and in-person advertisements, and booths at student residences and welcoming events. Incentives offered for participation included a free coffee voucher and entry into a draw to win an iPad. Student participants were provided a letter of information outlining the study aims and potential risks and benefits to participation, and they were provided consent to access and complete the survey.

It should be noted that nonsuicidal self-harm was collected using the C-SSRS [25], while item 9 of the PHQ-9 questionnaire [11,12] refers to thoughts of being better off dead or hurting oneself. Except otherwise stated, we will consider self-harm as the C-SSRS measure (self-harm using C-SSRS and item 9 of the PHQ-9 in different decision nodes).

**Table 1:** List of recreational drug use variables.

Variable description	Response options	Threshold used in rules
Nonprescribed sleeping pills, past month	0 = Never, 1 = Less than once a week, 2 = Once a week, 3 = 2-3 times a week, 4 = 4+ times a week, and 999 = prefer not to answer	≥2
Nonprescribed stimulants or wake-up pills, past month	0 = Never, 1 = Less than once a week, 2 = Once a week, 3 = 2-3 times a week, 4 = 4+ times a week, and 999 = prefer not to answer	≥2
Cannabis, past month	The first 2 terms defined options as: 0 = Never, 1 = Less than once a week, 2 = Once a week, 3 = 2-3 times a week, 4 = 4+ times a week, and 999 = prefer not to answer For other terms: 0 = Never, 1 = Less than once a month, 2 = 1-3 days a month, 3 = 1-2 days a week, 4 = 3-4 days a week, and 5 = Every day or nearly every day	≥2 (first 2 terms) ≥3 (other terms)
Pain killers or opiates, past month	0 = Never, 1 = Less than once a week, 2 = Once a week, 3 = 2-3 times a week, 4 = 4+ times a week, and 999 = Prefer not to answer	≥2
Psychedelics, past month	0 = Never, 1 = Less than once a week, 2 = Once a week, 3 = 2-3 times a week, 4 = 4+ times a week, and 999 = Prefer not to answer	≥2
Cocaine, past month	0 = Never, 1 = Less than once a month, 2 = 1-3 days a month, 3 = 1-2 days a week, 4 = 3-4 days a week, and 5 = Every day or nearly every day	≥3
Other street drugs (eg, opioids, LSD^a^, MDMA^b^)	0 = Never, 1 = Less than once a month, 2 = 1-3 days a month, 3 = 1-2 days a week, 4 = 3-4 days a week, and 5 = Every day or nearly every day	≥3
Prescription drug without a prescription to get high, buzzed, or numbed out	0 = Never, 1 = Less than once a month, 2 = 1-3 days a month, 3 = 1-2 days a week, 4 = 3-4 days a week, and 5 = Every day or nearly every day	≥3
Nonprescribed medication to enhance academic performance (eg, modafinil and stimulant medication)	0 = Never, 1 = Less than once a month, 2 = 1-3 days a month, 3 = 1-2 days a week, 4 = 3-4 days a week, and 5 = Every day or nearly every day	≥3
Binge drinking, 5+ alcoholic drinks on one occasion	0 = Never, 1 = Less than monthly, 2 = Monthly, 3 = Weekly, and 4 = Daily or almost daily	≥3

^a^LSD: lysergic acid diethylamide.

^b^MDMA: methylenedioxymethamphetamine.

**Table 2:** A priori set of features.

Variable names		Description
**PHQ-9^a^** **Items: Over the past 2 weeks, how often have you been bothered by the following problems: 0 = Not at all, 1 = Several days, 2 = Over half the days, and 3 = Nearly every day?**		
	PHQ9_1_1	Little interest or pleasure in doing things
	PHQ9_2_1	Feeling down, depressed, or hopeless
	PHQ9_3_1	Trouble falling or staying asleep or sleeping too much
	PHQ9_4_1	Feeling tired or having little energy
	PHQ9_5_1	Poor appetite or overeating
	PHQ9_6_1	Feeling bad about yourself, or that you are a failure, or have let yourself or your family down
	PHQ9_7_1	Trouble concentrating on things, such as reading the newspaper or watching TV
	PHQ9_8_1	Moving or speaking so slowly that other people could have noticed? Or the opposite—being so fidgety or restless that you have been moving around a lot more than usual?
	PHQ9_9_1	Thoughts that you would be better off dead or of hurting yourself in some way
	PHQ9_DIFF_1	If checked off any problems (PHQ-9), how difficult have these made for you to do your work, take care of things at home, or get along with other people? 0 = Not at all difficult, 1 = Somewhat difficult, 2 = Very difficult, and 3 = Extremely difficult
**GAD-7^b^** **Items: Over the past 2 weeks, how often** **have you** **been bothered by the following problems: 0= Not at all, 1= Several days, 2= Over half the days, and 3 = Nearly every day?**		
	GAD7_1_1	Feeling nervous, anxious, or on edge
	GAD7_2_1	Not being able to stop or control worrying
	GAD7_3_1	Worrying too much about different things
	GAD7_4_1	Trouble relaxing
	GAD7_5_1	Being so restless that it is hard to sit still
	GAD7_6_1	Becoming easily annoyed or irritable
	GAD7_7_1	Feeling afraid as if something awful might happen
	GAD7_DIFF_1	If checked off any problems (GAD-7), how difficult have these made for you to do your work, take care of things at home, or get along with other people? 0 = Not at all difficult, 1 = Somewhat difficult, 2 = Very difficult, and 3 = Extremely difficult
**Disorders: Have you ever been diagnosed with any of the following mental health conditions or learning difficulties? 1 = Yes; 2 = No**		
	ANXIETY_1	Anxiety disorder (eg, PTSD^c^, OCD^d^, panic disorder, social anxiety disorder, and generalized anxiety disorder)
	MOOD_1	Mood disorder (eg, depression, dysthymia, and bipolar disorder)
	PSYCHOTIC_1	Psychotic disorder (eg, schizophrenia and drug-induced psychosis)
	EATING_1	Eating disorder (eg, bulimia nervosa, anorexia nervosa, and binge eating disorder)
	NEURO_1	Neurodevelopmental disorder (eg, autism spectrum)
	SUBSTANCE_1	Substance use disorder (eg, cannabis and alcohol)
	EMERGE_1	In your lifetime, have you ever visited a hospital emergency department or been admitted to a hospital for help with a mental health condition?
**Family history: Have any of your first-degree blood relatives (eg, biological parents or siblings) ever been diagnosed with any of the following mental health conditions or learning difficulties? 1 = Yes; 2 = No**		
	FANXIETY_1	Anxiety disorder (eg, PTSD, OCD, panic disorder, social anxiety disorder, and generalized anxiety disorder)
	FMOOD_1	Mood disorder (eg, depression, dysthymia, and bipolar disorder)
	FPSYCHOTIC_1	Psychotic disorder (eg, schizophrenia and drug-induced psychosis)
	FSUBSTANCE_1	Substance use disorder (eg, cannabis and alcohol)
**Abuse: In terms of difficult childhood experiences… 1 = Yes; 2 = No**		
	REJECTED_1	When you were a child or teenager was someone in your household very harsh, critical, or rejecting toward you?
	PHYSABUSE_1	When you were a child or teenager were you ever hit repeatedly with an implement (such as a belt or stick) or punched, kicked, or burnt by someone in the household?
	BULLYING_1	When you were a child or a teenager were you physically or verbally bullied or teased very badly by peers?
	SABUSE_1	When you were a child or teenager did you ever have any unwanted sexual experiences?
	PDEATH_1	Parental death before 10 years old (either parent)
**Suicidality: Thinking about your past, have you ever: 1 = Yes; 2 = No**		
	WISHDEAD_1	Wished you were dead or wished you could go to sleep and never wake up?
	SUICIDEA_1	Had thoughts about ending your life?
	SUICATMP_1	Made any suicide attempts?
	SELFHARM_1	Hurt yourself on purpose without trying to end your life?
**PSS-4^e^** **: Thinking about stress, please indicate in the last month, how often have you… 0 = Never, 1 = Almost never, 2 = Sometimes, 3 = Fairly often, and 4 = Very often**		
	STRESS_1_1	Felt that you were unable to control the important things in your life?
	STRESS_2_1	Felt confident about your ability to handle your personal problems?
	STRESS_3_1	Felt that things were going your way?
	STRESS_4_1	Felt difficulties were piling up so high that you could not overcome them?

^a^PHQ-9: 9-item Patient Health Questionnaire.

^b^GAD-7: 7-item Generalized Anxiety Disorder questionnaire.

^c^PTSD: posttraumatic stress disorder.

^d^OCD: obsessive-compulsive disorder.

^e^PSS-4: Perceived Stress Scale–4.

### Missing Data Imputation

Missing baseline variables were imputed using multivariate imputations by chained equations [26,27]. Ten multiple-imputed datasets were produced using a combination of predictive mean matching and polynomial regression models. For tools with individual item–level data (eg, individual questions from the GAD-7 and PHQ-9), the individual items were imputed using predictive mean matching, while the total scores were derived using passive imputation [26]. All features required for the ML models were included in the imputed variables, as well as variables collected as part of the U-Flourish study that may inform the imputation process that were not included in the ML model (eg, academic program of the student). A single-imputed dataset was then constructed for use in the ML models, using the mode for each imputed baseline variable. All imputation preparation and completion were performed using R (version 4.3.2: R Core Team) for Windows 64bit [28].

### Defining Stepped Care Levels

**Figure 1:**
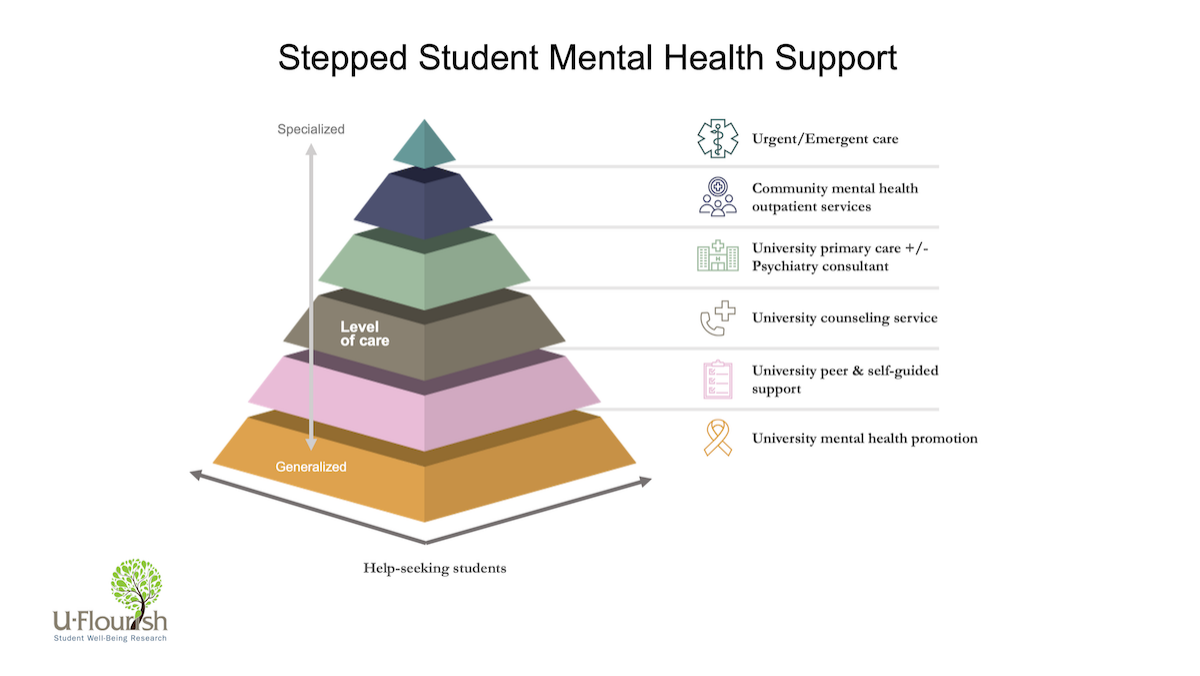
General stepped care framework.

Iterative co-design sessions were held biweekly between an interdisciplinary group of researchers and clinicians in health informatics, psychiatry, public health, nursing, and statistics. These sessions were initially used to define a consensus map of stepped care levels for the recommendation engine to address (Figure 1).

Of note, the stepped care levels were designed to be a general template that can be customized for any higher education institution and their respective mental health prevention and early intervention services. These university supports can then be classified within the general stepped care levels, which are based on the nature and intensity of the intervention.

Lower levels were defined as broader and less resource-intensive, emphasizing mental health promotion, psychoeducation, and self-guided support for managing common mental health problems (ie, insomnia, stress, and time management). Higher levels were defined as more resource-intensive and would typically involve in-person assessment and guided (supervised by a mental health professional) intervention.

### Prediction Models and Recommendation Engine Design and Development

**Figure 2:**
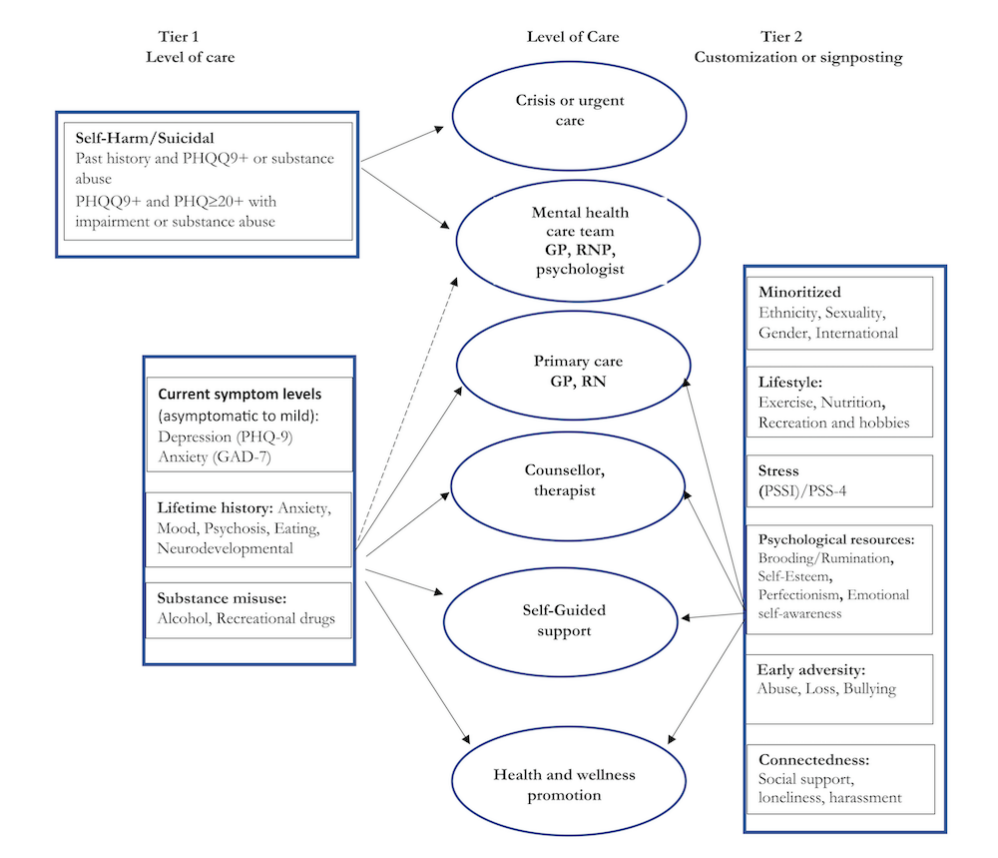
Mapping of a priori clinically relevant U-Flourish features. List of features that are important contributors to mental health outcomes and that could be used to inform high-level signposting to treatment recommendations (tier 1), and features that could be used to further personalize the recommendations (tier 2). GAD-7: 7-item Generalized Anxiety Disorder questionnaire; GP: general practitioner; PHQ-9: 9-item Patient Health Questionnaire; PSS-4: Perceived Stress Scale–4; PSSI: Secondary Student Stressors Index; RN: registered nurse; RNP; registered practical nurse.

After defining the stepped care levels, our biweekly co-design discussions focused on the design, development, and evaluation of the recommendation engine. First, we defined a priori features that were important contributors to mental health outcomes and that could be used to inform signposting to treatment recommendations. We also identified and described features that could be used for personalizing the treatment recommendations in the future (Figure 2). Then, as outlined in the following sections, two strategies were used to develop the engine: (1) we created a set of clinically defined rules, that is, a rules-based algorithm, to assess current mental health status and related level of treatment signposting; and (2) we developed ML-based models to predict future risk of worsening mental health for targeting mental health promotion and prevention.

Of note, tier 2 (right-hand side of Figure 2) is meant to provide more personalized filters based on individual preferences and characteristics (eg, international student, members of minority communities, and early adversity). While this level of customization is outside the scope of this work, we decided to include it as part of Figure 2 for future considerations.

### Rules-Based Algorithm for Categorizing Current Level of Treatment Needed

#### Overview of Levels

We sought consensus among the working group members—defined as agreement by all group members as to the best course of action—as to clinical decision points for clinically predictive variables at 2 workflow levels. The first level examines urgency, that is, establishing whether a student requires a timely in-person assessment. The second level provides less-intensive support recommendations based on depression and anxiety symptoms (level 2a), lifetime history (level 2b), and substance use (level 2c). Table 1 describes the variables used in the rules that consider drug use, while Table 2 describes the remaining variables used both in the rules and in the ML models. It should be noted that the ML models were developed in parallel with the rules.

#### Level 1: Urgency of Assessment and Support Workflow

Signposting to either more urgent and timely, in-person assessment and support, or less immediate support (ie, less intensive levels of assessment and support) was based on four self-report criteria: (1) whether a student had a lifetime history of a suicide attempt or self-harm, (2) whether the student had current suicidal thoughts and self-harm, (3) whether the student was experiencing current debilitating and severe depression, and (4) whether the student was currently using substances (alcohol, cannabis, or recreational drugs).

**Figure 3:**
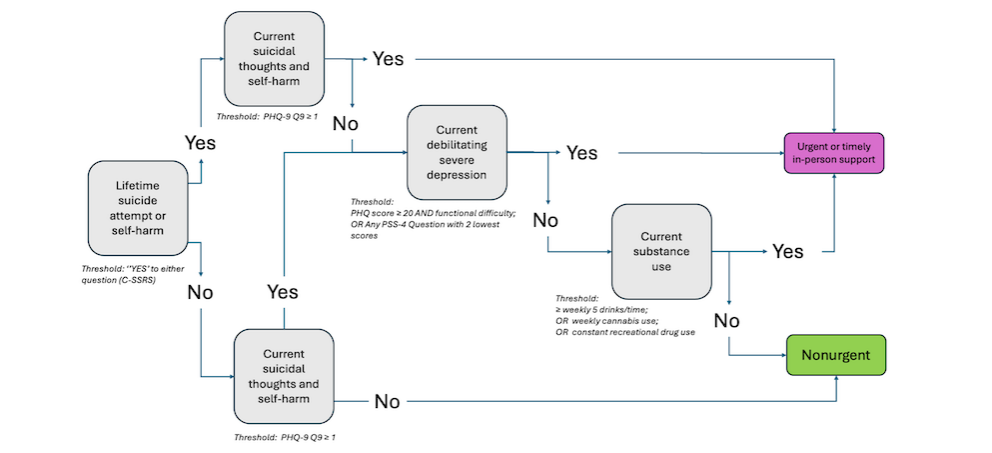
In-person urgent or timely level rules. Summary of the rules-based algorithm signposting students as either urgent (requiring more immediate or timely in-person assessment) or nonurgent (requiring less immediate follow-up, represented by rules in Figures 4-6). The algorithm shows the flow of decision points starting with a screen for lifetime suicide attempt or self-harm. If positive, it screens for current suicidal thoughts or self-harm. If negative, it screens for severe debilitating depression and current substance use. Aside from the first decision point (positive for lifetime suicide attempt), in the presence of any additional thresholds met, the student is signposted to requiring urgent intervention. C-SSRS: Columbia-Suicide Severity Rating Scale; PHQ-9: 9-item Patient Health Questionnaire; PSS-4: Perceived Stress Scale–4.

Figure 3 shows the flow of decision points starting with a screen for lifetime suicide attempt or self-harm (C-SSRS), followed by a screen for current suicidal thoughts or self-harm (PHQ-9 item 9). If lifetime attempt or self-harm is screened positive and current thoughts or self-harm are negative (score of 0 for PHQ-9 item 9), or if the opposite is true (ie, lifetime attempt or self-harm is negative and current thoughts or self-harm is positive), the rule considers presence of severe depression and substance misuse. Aside from these decision points, in the presence of any additional thresholds met the student is signposted to requiring a more timely and in-person assessment and intervention.

#### Level 2: Treatment Recommendation–Level Workflow

##### Overview of Level 2

All students who were classified as not requiring urgent or timely in-person assessment enter the treatment recommendation workflow which considers each of depression and anxiety symptoms (level 2a), lifetime history (level 2b), and substance use (level 2c). This process allows for robust and nuanced signposting of students to each of the stepped care levels outlined in Figure 1. These 3 sets of rules (ie, considering first by symptom, lifetime history, or substance use) are evaluated in parallel (ie, an individual could be screened for different sets of rules at the same time); in this case, the signposting should be the highest level of treatment recommended by the rules.

##### Level 2a: Presence of Depressive and Anxiety Symptoms

**Figure 4:**
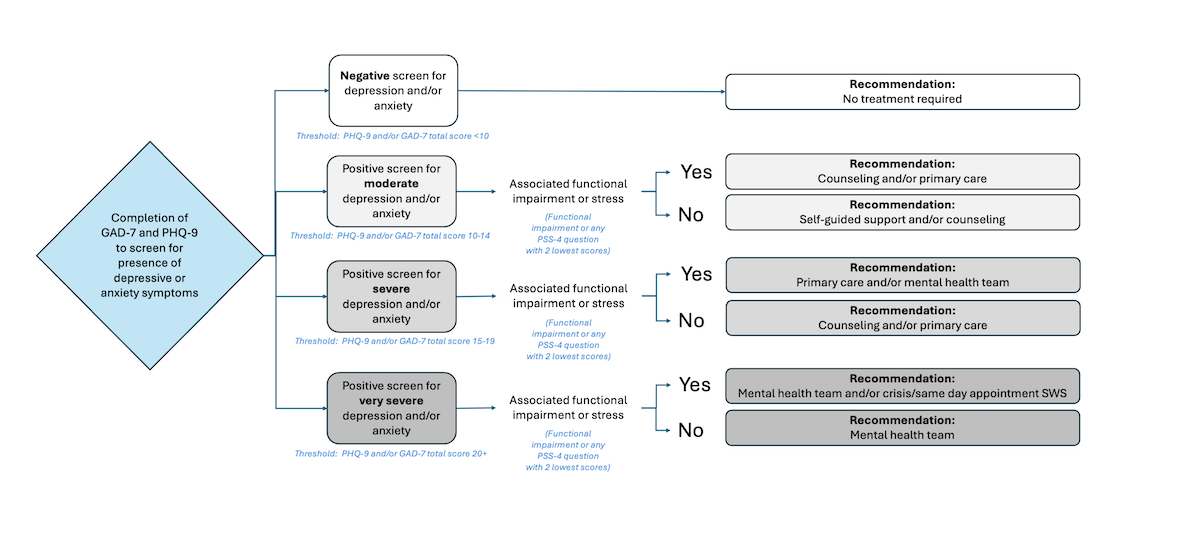
Rules-based algorithm—symptoms of depression and anxiety rules. Summary of the rules-based algorithm signposting students based on symptoms of depression and anxiety. Depending on the level of depressive or anxious symptoms (moderate, severe, or very severe) and presence of associated functional impairment or stress, individuals are signposted varied levels of stepped care from lower intensity (eg, self-guided support and counseling) to higher intensity (eg, mental health team and crisis/same day appointment Student Wellness Services). GAD-7: 7-item Generalized Anxiety Disorder questionnaire; PHQ-9: 9-item Patient Health Questionnaire; PSS-4: Perceived Stress Scale–4; SWS: Student Wellness Services.

Figure 4 shows the decision nodes of level 2a. Recommendations were based on the combination of severity of depressive and anxiety symptoms defined from the PHQ-9 and the GAD-7, paired with associated functional impairment (asked after the PHQ-9 and GAD-7 questionnaires, as seen in Table 2) or stress defined from the PSS-4 (having any PSS-4 question achieving the lowest scores, that is, score of 3 or higher for questions 1 and 4, and score of 1 or lower for questions 2 and 3). Cutoffs were defined by the experts on the team.

##### Level 2b: Presence of Depressive and Anxiety Symptoms

**Figure 5:**
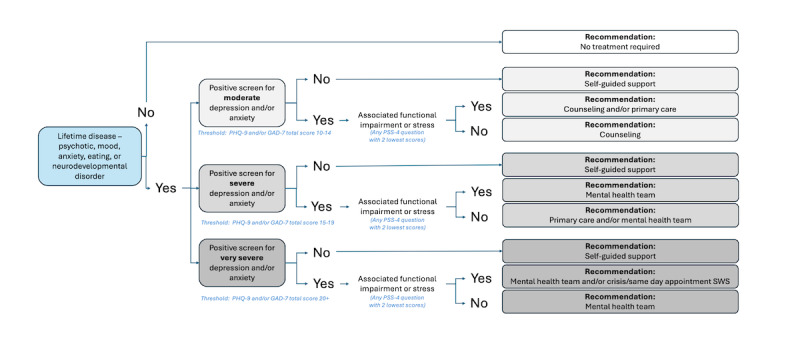
Rules-based algorithm—lifetime history rules. Summary of the rules-based algorithm signposting students based on symptoms of depression and anxiety and presence of lifetime conditions. Depending on the presence of these lifetime conditions, the level of depressive or anxious symptoms (moderate, severe, or very severe), and presence of associated functional impairment or stress, individuals are signposted to varied levels of stepped care from lower intensity (eg, self-guided support) to higher intensity (eg, mental health team and crisis/same day appointment Student Wellness Services). GAD-7: 7-item Generalized Anxiety Disorder questionnaire; PHQ-9: 9-item Patient Health Questionnaire; PSS-4: Perceived Stress Scale–4; SWS: Student Wellness Services.

Figure 5 shows the decision nodes of level 2b. To account for additional nuances of those with a lifetime history of diagnoses, an additional layer of self-guided support was added compared with level 2a. In addition to a lifetime diagnosis history (psychotic, mood, anxiety, eating, or neurodevelopmental disorders), recommendations were based on the combination of severity of screening positive for depression and anxiety defined from the PHQ-9 and the GAD-7, paired with associated functional impairment or stress (similarly to level 2a).

##### Level 2c: Presence of Depressive and Anxiety Symptoms in Those Who Use Substances

**Figure 6:**
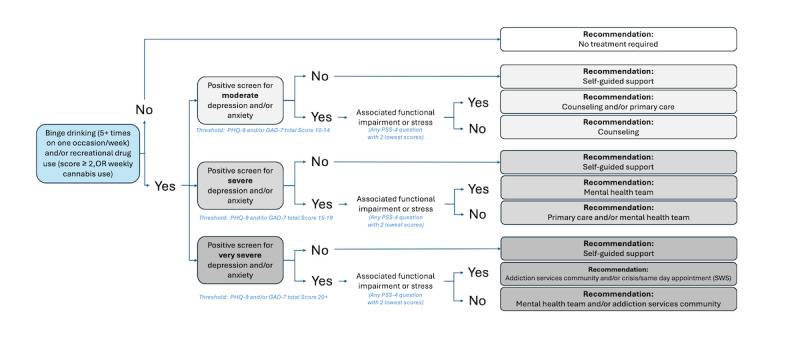
Rules-based algorithm—substance abuse rules. Summary of the rules-based algorithm signposting students based on symptoms of depression and anxiety and substance abuse. Depending on the presence of substance abuse, the level of depressive or anxious symptoms (moderate, severe, or very severe), and presence of associated functional impairment or stress, individuals are signposted to varied levels of stepped care from lower intensity (eg, self-guided support) to higher intensity (eg, addiction services in community and crisis/same day appointment Student Wellness Services). GAD-7: 7-item Generalized Anxiety Disorder questionnaire; PHQ-9: 9-item Patient Health Questionnaire; PSS-4: Perceived Stress Scale–4; SWS: Student Wellness Services.

Figure 6 shows the decision nodes of level 2c. As in level 2b, to account for additional nuances of those with a lifetime history of diagnoses, the same additional layer of self-guided support was included for those who use substances (ie, 5+ drinks on at least 1 occasion per week) or use recreational drugs (score ≥2 for any drug, or weekly cannabis use).

### ML-Based Models for Mental Health Worsening Risk Prediction

The clinically defined rules assess current mental health status. We also sought to predict future risk of screening positive for clinically significant anxiety and depressive symptoms, that is, for those who might be at risk in the future but screen negative at baseline—through the development of ML models. In particular, we trained the XGBoost model with a subset of U-Flourish features identified a priori by clinicians during the iterative sessions as model inputs (Table 2). As a reminder, these features were collected upon school entry, in the Fall term, that is, time 1 (Table 2), and included self-report indicators of depression (PHQ-9), anxiety (GAD-7), lifetime diagnosis or family history of diagnoses (anxiety, mood, psychotic, disordered eating, and neurodevelopmental conditions), personal and family history of substance use, childhood adversity (ie, abuse, neglect, or death of a parent), history of suicidality or self-harm, and current stress (Multimedia Appendix 1). This process was repeated for the PHQ-2 and GAD-2 questionnaires to evaluate whether these brief 2-item measures could be used to successfully predict depression or anxiety, without the remaining PHQ-9 and GAD-7 items, respectively. In other words, models using the PHQ-2 and the GAD-2 contained the first 2 items of each questionnaire in the model input to support more frequent collection of data points without increasing respondent burden.

### Defining Thresholds for Output Labels

To label students as having clinically significant levels of anxiety or depression, a standard cutoff score of 10+ for each of the GAD-7 and PHQ-9 questionnaires was used [11,29-32]. Model outputs were created to predict anxiety and depression symptom scores measured by the GAD-7 and the PHQ-9 at the end of the academic year (ie, time 2: before Spring term examinations). In other words, if a student scored 10 or above on the PHQ-9 or GAD-7 at time 2, they were labeled as having a positive screen for depression or anxiety, respectively.

### Classification Metrics

Binary classification was performed on the labeled data and the metrics of accuracy, precision, recall, and *F*_1_-score were calculated to assess prediction performance. Accuracy was defined as the proportion of correct predictions compared with the total predictions. Precision was defined as the proportion of true-positive predictions (ie, if an individual is clinically anxious or depressed) and was used to indicate positive predictive value (PPV) and negative predictive value (NPV) as follows:

Precision:

- PPV: True positives / (True positives + False positives)
- NPV: True negatives / (True negatives + False negatives)

We used recall to indicate sensitivity and specificity. Sensitivity was defined as the proportion of true-positive predictions among all actual positives. Conversely, specificity was defined as the proportion of true-negative predictions among all actual negatives.

Recall:

- Sensitivity: True positives / (True positives + False negatives)
- Specificity: True negatives / (True negatives + False positives)

Finally, *F*_1_-score is a typically used metric to evaluate ML models and represent the harmonic mean between precision and recall, calculated as follows:

*F*_1_-score: 2 × (Precision × Recall) / (Precision + Recall)

Due to class imbalance (ie, having more examples of the negative class than the positive class), the *F*_1_-macro metric was given priority, as it considers all classes as having equal weights when calculating the score and avoids artificially inflated results due to predictions from one class severely outperforming the other [33,34]. Libraries used include sci-kit learn and Python’s XGBoost implementation.

### Training and Testing

The datasets for ML training consisted of 2285 people who completed the U-Flourish survey in the Fall between 2018 and 2022, were not signposted to an in-person assessment (timely or urgent), were not screened in the level 2a rules, and had survey data for the following Spring of the academic year 2019-2023.

The dataset was split into train and test sets using a 70:30 ratio, stratified by clinical outcome (ie, anxiety and depression). For the depression outcome, the dataset’s distribution had an imbalance: from1806 samples screened negative for depression to only 479 screening positive (1:3.77 ratio). Anxiety displayed a similar imbalance consisting of 1792 individuals not screening positive for anxiety compared with 493 screening positive (1:3.63 ratio). To alleviate this imbalance on model training, we included “scale_pos_weight” as part of XGBoost’s training parameter, which scales the punishment of errors in predicting the minority class, causing the model to overcorrect. Values tested for this parameter were 1 (indicating the new parameter does not scale weights), followed by proportions based on the ratio of negative instances divided by positive instances, following best practices listed in the XGBoost’s documentation for the general use of the parameter [35]. Finally, we evaluated and fine-tuned the model through 10-fold Cross-Validation through sci-kit learn’s StratifiedKFold function.

### Feature Importance

We used the Shapley additive explanations (SHAP) method [36] to investigate feature importance (ie, which features had the most influence on our model’s predictions). SHAP gives each feature a score, called a “Shapley score,” that shows how important that feature is for making accurate predictions [36]. To visualize the results, we used a SHAP summary plot that displays how each feature affects the model. Specifically, each point represents a sample from our data. The position of the point on the x-axis shows the feature’s effect on the prediction (with positive values pushing toward a positive result and negative values toward a negative one). The color of each point represents the feature’s value, with red indicating higher values and blue indicating lower ones. This setup aids in identifying not only which features are important but also how they interact with the model’s output [36].

### Comparative Analysis and Optimizations

To clinically assess the quality of the ML models, we conducted a comparative analysis between models using inputs from the PHQ-9, PHQ-2, GAD-7, and GAD-2, specifically evaluating their sensitivity (recall of the positive class), specificity (recall of the negative class), PPV, and NPV as the comparison metrics. As confirmed by clinicians during working meetings, there is no set “gold standard” target for these metrics in university students. We therefore defined our internal targets based on literature investigating clinical use of the PHQ and GAD questionnaires in student and general adult populations (Tables 3 and 4).

**Table 3:** Metrics based on student populations.

	Sensitivity (recall 1)	Specificity (recall 0)	PPV^a^ (precision 1)	NPV^b^ (precision 0)
PHQ-9^c^ [11-13,29,37-40]	83%	83%	65%	95%
GAD-7^d^ [13,14]	84%	65%	60%	92%
PHQ-2^e^ [41]	N/A^f^	N/A	N/A	N/A
GAD-2^g^ [42]	94%	86%	65%	98%

^a^PPV: positive predictive value.

^b^NPV: negative predictive value.

^c^PHQ-9: 9-item Patient Health Questionnaire.

^d^GAD-7: 7-item Generalized Anxiety Disorder questionnaire.

^e^PHQ-2: 2-item Patient Health Questionnaire.

^f^N/A: not applicable.

^g^GAD-2: 2-item Generalized Anxiety Disorder Questionnaire.

**Table 4:** Metrics based on the general population.

	Sensitivity (recall 1)	Specificity (recall 0)	PPV^a^ (precision 1)	NPV^b^ (precision 0)
PHQ-9^c^ [29,31,43-65]	80%	84%	39%	96%
GAD-7^d^ [24,32,64-68]	71%	80%	29%	99%
PHQ-2^e^ [29,40,48,50,51,60,62,65,69-74]	75%	79%	53%	88%
GAD-2^f^ [32,51,64-66,68,75-77]	76%	75%	62%	88%

^a^PPV: positive predictive value.

^b^NPV: negative predictive value.

^c^PHQ-9: 9-item Patient Health Questionnaire.

^d^GAD-7: 7-item Generalized Anxiety Disorder questionnaire.

^e^PHQ-2: 2-item Patient Health Questionnaire.

^f^GAD-2: 2-item Generalized Anxiety Disorder Questionnaire.

The aforementioned metrics were used to examine the trade-off between false positives and negatives, as the main goal of the models was to minimize the false-negative predictions. In other words, we paid close attention to how many false positives should be allowed, while simultaneously minimizing false negatives through iterative consultation with clinician experts on the team. The final goal of the model was to signpost students to appropriate treatment recommendations using their self-report data within a stepped care approach (ie, not to provide clinical triage). We used the metrics from Table 3 and 4 as a guide to define the minimum sensitivity that the system needs based on how these questionnaires are used in the literature.

In this context, we explored adjusting the probability threshold used to classify observations. Instead of the usual 50% cutoff, we tested different thresholds to match sensitivity levels reported in Tables 3 and 4. We used the *yellowbrick* [78] library to visualize how different thresholds affect precision, recall, and the number of true and false positives or negatives. Such numbers were validated with clinicians during the iterative discussion sessions.

### Integrating the ML Models Into the Recommendation Engine

**Figure 7:**
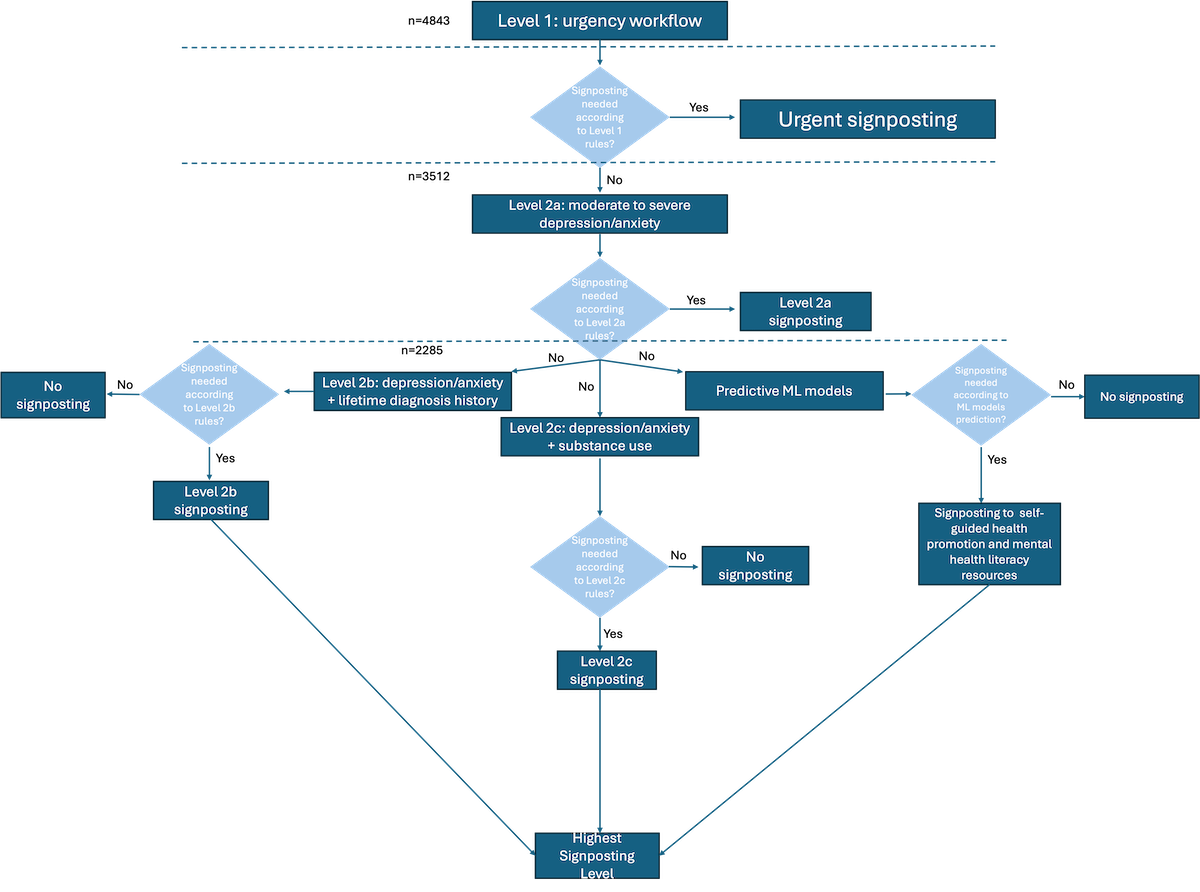
Recommendation engine—flow of rules and machine learning models for signposting to treatment recommendations. Summary of the recommendation engine’s flow of rules and machine learning (ML) models. First, students (n=4843) are screened based on the level 1 rule (Figure 3). If they meet the level 1 rule criteria, they are signposted to urgent or timely in-person assessment. If not, they are first screened by level 2a rule (n=3512). If they meet the level 2a rule criteria, they are signposted to a treatment recommendation based on the symptoms displayed (Figure 2a). If not, they (n=2285) are screened in parallel by the level 2b rule (Figure 5), level 2c rule (Figure 6), and by the ML model that predicts future risk. Each of these will generate a signposting to a treatment recommendation, the highest of which will be selected. ML: machine learning.

Some students may not be currently experiencing clinically significant symptoms of anxiety and depression, but could be at future risk of developing said symptoms. The clinically defined rules capture students *currently* experiencing mental health challenges. The ML models predicts *future* risk of worsening mental health. As such, the clinically defined rules and ML models offer 2 complementary approaches to prediction models which are integrated into the recommendation engine as follows (Figure 7).

First, students are evaluated according to the level 1 rules (urgency-level workflow). The highest level of signposting requires timely in-person assessment. Students who do not meet the urgency workflow criteria are evaluated on level 2a (presence of depressive and anxiety symptoms). Levels 1 and 2a screen for immediate risk (eg, suicidality) as well as moderate to severe levels of anxiety and depression; if students do not meet these thresholds, they are then evaluated according to level 2b (presence of depressive and anxiety symptoms in those with lifetime diagnosis history), level 2c (presence of depressive and anxiety symptoms in those who use substances), and the predictive ML models in parallel.

### Ethical Considerations

The U-Flourish study was reviewed for ethical compliance and approved by the Queen’s University and Affiliated Teaching Hospitals Research Ethics Board (HSREB PSIY-609-18). Informed consent was obtained from all participants, who are also made aware that participation is voluntary and does not impact academic standing. Students completing the Fall surveys receive a free coffee or tea valued at CAD $2 (US $1.45) from the local campus serving to be redeemed every Friday over the month the survey is running by showing proof of survey completion to the cashier. At the completion of both annual surveys (Fall and Spring), students are entered into a draw to win 1 of 5 iPads, valued at CAD $450 (US $325). Only deidentified data are shared.

## Results

### Descriptive Analyses

Table 5 provides the sample demographics of the total 4843 individuals in the dataset, the remaining 3512 individuals who were not screened by level 1 rules, and the 2285 individuals who were part of the ML models.

Consistently, they were predominantly female and white, aged between 16 and 20 years. When looking at the total population (n=4843), the PHQ-9 score had a mean PHQ-9 value of 7.72 (SD 5.92; scores range from 1 to 27) and mean GAD-7 value of 8.01 (SD 5.55; scores range from 1 to 21). These scores decrease per group as expected, since each group moves from higher need of assessment to lowest.

**Table 5:** Sample demographics.

Characteristics			Participant groups				
			In the dataset (n=4843)		Not screened by level 1 rules (n=3512)		Part of the ML models (n=2285)
**Score, mean (SD)**							
	PHQ-9^a^	7.72 (5.92)		6.00 (4.72)		3.48 (2.56)	
	GAD-7^b^	8.01 (5.55)		6.92 (5.09)		3.96 (2.65)	
**Age (years), n (%)**							
	16-20	4552 (94)		4552 (94)		2125 (93)	
	21-25	242 (5)		242 (5)		114 (5)	
	26+	49 (1)		49 (1)		46 (2)	
**Sex, n (%)**							
	Male	1293 (26.7)		1011 (28.8)		779 (34.1)	
	Female	3477 (71.8)		2469 (70.3)		1499 (65.6)	
	Nonbinary	53 (1.1)		25 (0.7)		5 (0.2)	
	Prefer not to say	20 (0.4)		7 (0.2)		2 (0.1)	
**Ethnicity, n (%)**							
	White	3438 (71)		2494 (71)		1622 (71)	
	East/Southern Asian	1017 (21)		702 (20)		480 (21)	
	South Asian	339 (7)		246 (7)		137 (6)	
	Black	145 (3)		105 (3)		69 (3)	
	Latin	97 (2)		70 (2)		46 (2)	
	Indigenous	97 (2)		35 (1)		23 (1)	
	Middle Eastern	194 (4)		141 (4)		69 (3)	
	Other	48 (1)		35 (1)		23 (1)	
**International status, n (%)**							
	International student	436 (9)		421 (12)		201 (9)	

^a^PHQ-9: 9-item Patient Health Questionnaire.

^b^GAD-7: 7-item Generalized Anxiety Disorder Questionnaire.

### Rule-Based Algorithm for Categorizing Current Level of Treatment Needed

#### Overview of Levels

This section describes the results of validating the rules-based algorithm on the entire dataset. As described in the previous section, this algorithm entails 2 levels: level 1, which signposts to in-person urgent or timely level assessment, and level 2 comprising nonurgent assessments (this level is also divided into 3 rules and used to signpost students to treatment recommendations based on a stepped care approach, as seen in Figures 4-6). Of note, this study was meant to determine how the algorithms and models would behave with real student data; individual student data were deidentified and students were not contacted about recommendations.

#### Level 1: In-Person Urgent or Timely Level Rules

Out of a total of 4843 people, 1331 (27.5%) were recommended to the timely assessment or urgent care levels based on the application of these rules. The remaining 3512 individuals were then evaluated by the other rules (symptom-based, lifetime history, and substance misuse).

#### Level 2: Nonurgent Recommendation–Level Rules

##### Level 2a

Out of the 3512 individuals, 797 (22.7%) were signposted with moderate symptoms, 358 (10.2%) with severe symptoms, and 72 (2%) with very severe symptoms. A total of 2285 (65%) individuals were not signposted, that is, did not display at least moderate symptoms of anxiety or depression, and were thus eligible for the ML aspect of the recommendation system. In other words, such individuals are not at current risk based on anxiety or depressive symptoms but will still be assessed for future risk. As a reminder, an individual can be eligible for both the ML system and still be assessed by the lifetime history and substance misuse rules (levels 2b and c).

Out of the 1227 individuals signposted to a stepped care level, 53 (1.5%) were signposted to self-guided support and counseling, 753 (21.4%) to counseling and primary care, 349 (9.9%) to primary care and mental health team, 1 (0.02%) to mental health team, and 71 (2%) to mental health team and crisis/same day appointment with wellness services.

##### Level 2b

In total, 7.2% (253/3512) of individuals are signposted with moderate symptoms, 4.2% (146/3512) with severe symptoms, and 1.1% (39/3512) with very severe symptoms. In total 11.2% (394/3512) of individuals are signposted based on their lifetime history alone without associated screen positives for depression or anxiety. In total, 76.3% (2680/3512) of individuals were not signposted.

Out of the 832 individuals signposted to a stepped care level, 394 (11.2%) were signposted to self-guided support, 27 (0.8%) to counseling, 226 (6.4%) to counseling and primary care, 4 (0.1%) to primary care and mental health team, 143 (4.1%) to mental health team, and 38 (1.1%) to mental health team and crisis/same day appointment with wellness services.

##### Level 2c

For this level, we have the largest number of individuals signposted based on substance use alone (1674/3512, 47.7%). The great majority of these, 1147 individuals, were signposted due to reporting binge drinking behavior (5 drinks or more per week on 1 occasion). In total, 14.6% (511/3512) are signposted based on moderate screen, 6% (211/3512) with severe screens for anxiety or depression, and 1.6% (56/3512) with very severe screens. In total, 30.2% (1060/3512) of individuals are not signposted.

Out of the 2452 individuals signposted, the 1674 individuals (47.7%) screened due to binge drinking were signposted to self-guided support, 25 (0.7%) to counseling, 486 (13.8%) to counseling and primary care, 5 (0.1%) to primary care and mental health team, 206 (5.9%) to mental health team, 1 (0.03%) to mental health team and addiction services community, and 55 (1.6%) to addiction services community and crisis/same day appointment with wellness services.

### ML Model

As previously mentioned, the ML models were created using the XGBoost algorithm, taking as input a priori features (Table 2). We show the results of the ML models for predicting anxiety or depression, using PHQ-9, GAD-7, PHQ-2, and GAD-2 as inputs in Table 6. All models had sensitivity of 90% or above, aligning with Tables 3 and 4.

More details on the contingency table and evaluation metrics can be seen in Tables S1 through S8 in Multimedia Appendix 1. We call 0 or 1 the class labels representing the negative and positive classes, respectively. In the context of anxiety, these labels represent someone not having clinically significant anxiety, that is, GAD-7 score less than 10 (negative class), or someone having clinically significant anxiety, that is, GAD-7 score of 10 or more (positive class). In the context of depression, they represent someone not having clinically significant depression, that is, PHQ-9 score less than 10 (negative class), or someone having clinically significant depression, that is, PHQ-9 score of 10 or more (positive class).

The final prediction probability threshold for the GAD-7 model was 14%, as opposed to the usual cutoff of 50%. The threshold for the other models was 11%. This was done to adjust the sensitivity to align with Tables 3 and 4; in other words, we reduced the number of false negatives by increasing the sensitivity of the model, which also increased the number of false positives (decreasing the specificity). The optimal “scale_pos_weight” parameter for all models was set to 1, indicating that there was no scaling in the weight of errors predicting the minority class.

**Table 6:** Results of machine learning models.

	Sensitivity (recall 1)	Specificity (recall 0)	PPV^a^ (precision 1)	NPV^b^ (precision 0)
PHQ-9^c^	94%	18%	24%	93%
GAD-7^d^	90%	24%	24%	90%
PHQ-2^e^	90%	20%	23%	89%
GAD-2^f^	92%	19%	43%	90%

^a^PPV: positive predictive value.

^b^NPV: negative predictive value.

^c^PHQ-9: 9-item Patient Health Questionnaire.

^d^GAD-7: 7-item Generalized Anxiety Disorder questionnaire.

^e^PHQ-2: 2-item Patient Health Questionnaire.

^f^GAD-2: 2-item Generalized Anxiety Disorder Questionnaire.

### Feature Importance of ML Models

We used a SHAP summary plot to understand how different feature values influence the model’s predictions. Refer to Table 2 for feature naming, definitions, and scoring. The following sections detail the results of the feature importance analysis.

#### Anxiety

##### GAD-7 Model

We found that items 3 and 5 of the GAD-7, lifetime anxiety disorder, item 3 of the PSS-4, and a family history of mood disorder were associated with a greater likelihood of screening positive for clinically significant anxiety at time 2. Looking beyond these top 5 features, we can see that several PHQ-9 items, additional GAD-7 and PSS-4 items, family history of anxiety disorder, functional impairment, and diagnosis of disorders (eg, eating and psychotic) also contribute to the prediction. Figure S1 in Multimedia Appendix 1 shows the SHAP summary plot for the GAD-7 model.

##### GAD-2 Model

This model takes only the first 2 GAD-7 items as input rather than the entire questionnaire. Lifetime history of anxiety is the most important feature of this model, followed by item 3 of the PSS-4, item 2 of the GAD-7, family history of anxiety, and item 4 of the PSS-4. When looking into the importance of other features, we see that a history of abuse (most prominently bullying), functional impairment, other disorders such as mood and eating, self-harm, and additional items of PSS-4 and PHQ-9 are all important contributors to the prediction. Figure S2 in in Multimedia Appendix 1 shows the SHAP summary plot for the GAD-2 model

#### Depression

##### PHQ-9 Model

PHQ-9 items 3, 5, and 9 appear in the top 5 most important features and contribute toward a positive depression prediction. A family history of mood disorders, as well as item 4 of the PSS-4, is the remaining feature in the top 5. Other PHQ-9 items appear prominently as top contributors, as well as functional impairments. Like the GAD-2 model, bullying seems to contribute to clinically significant depression risk. GAD-7 items also appear as important predictors, indicating that anxiety and depression are highly correlated. Additional PSS-4 items, abuse, family history of disorders, and self-harm are all important predictors of this model. Figure S3 in Multimedia Appendix 1 shows the SHAP summary plot for the PHQ-9 model.

##### PHQ-2 Model

When changing the input to include only the first 2 items of the PHQ-9 and GAD-7, the topmost feature becomes the first PSS-4 item, followed by early experiences with sexual abuse, item 2 of the GAD-7, a visit to an emergency department due to a mental health condition, and functional impairment associated with depression. In addition to these features, additional PSS-4 items, family history of mental disorders (mood in particular), history of abuse (eg, bullying or rejection), suicide attempt, self-harm, and disorder diagnosis (eg, psychotic) also influence the prediction. Figure S4 in Multimedia Appendix 1 shows the SHAP summary plot for the PHQ-2 model.

## Discussion

### Principal Findings

This paper summarizes the development of a mental health calculator and support recommendation engine tailored for undergraduate students. U-Flourish longitudinal student survey data were used to develop the prediction models and corresponding recommendation engine. The prediction models, developed as part of the recommendation engine, were created using a hybrid approach, leveraging clinically defined rules and predictive models to improve early detection and accurate signposting. Findings from this study form the first major step in development of a digital application to provide accurate and useful feedback to students about their mental health, currently and over the course of the year, and to suggest indicated early intervention and prevention resources based on data they share. The aim is to improve prevention and early intervention of common mental health concerns and rationalize service utilization, using the 2 approaches of clinically defined rules and ML models, to assess current mental health status and predict future risk, respectively.

Main findings included that just more than 25% of students met the clinically defined rules for probable anxiety or depression and were signposted to in-person assessment through urgent, crisis, or student health primary care. Furthermore, ML models adjusted for optimal sensitivity were able to successfully predict the risk of students developing clinically significant anxiety or depression over the academic year. The most important predictors of these models included current depressive and anxiety symptoms, stress, early history of abuse, and family history of disorders. We showed that the use of the first 2 items of PHQ-9 and GAD-7 (PHQ-2 and GAD-2, respectively) can successfully predict risk, when coupled with other features, indicating that the full questionnaire data do not need to be collected for successful predictions, reducing the student burden.

### Rule-Based Algorithm

Out of all students, 27.5% were considered appropriate for a timely or urgent signposting level for further assessment based on the level 1 workflow. When considering the next tier of less immediate recommendations (level 2a, Figure 4), more people were filtered by *moderate* depression and anxiety (797 individuals) than severe or very symptom levels (430 individuals). This is expected from a clinical standpoint, as each category in level 2a progressively evaluates higher levels of reported anxiety and depression. In general, the results reveal that the rules are consistent with other data and patterns in student mental health, which further support the validity of the rule-based algorithm.

In addition, when considering the substance use rules, almost 50% of students were signposted to treatment recommendations on the basis of substance use alone, without any associated mental health symptoms, specifically binge drinking (defined as 5 drinks on 1 occasion, weekly). This is consistent with other Canadian data on university students [79] and points to a concerning culture of alcohol abuse among undergraduate students.

### ML Models

Four ML models were developed, considering as inputs the PHQ-9, GAD-7, PHQ-2, and GAD-2 (in addition to the other inputs), each predicting clinically significant depression or anxiety at time 2. When looking into the models, optimized also by probability classification threshold, results using only PHQ-2 and GAD-2 items as inputs were comparable with results from models using the complete questionnaires (Table 6) in terms of sensitivity. These results suggest that models trained on the abbreviated questionnaires can be used for successful prediction of depression and anxiety rather than relying on the full versions.

Of note, during the cross-validation process, the optimal models indicated that weight scaling was not needed. This suggests that, while there is a class imbalance, the ratio from majority to minority classes was not large enough to require a weighted correction. As previously discussed, we explored different probability thresholds as opposed to the traditionally used 50% cutoff of an item belonging to a certain class, in order to achieve a target sensitivity similar to the ones in Tables 3 and 4. The thresholds between all models were similar, with 0.14 for the GAD-7 and 0.11 for the other models.

With these adjustments, all models had a sensitivity higher than the ones obtained in the literature for Table 3, except the model that takes GAD-2 as input—with model sensitivity of 92% compared with the sensitivity of 94% in Table 3. However, with the exception of the PHQ-9 model, few studies among more than 40 peer-reviewed papers applied questionnaires specifically to student populations. In particular, only 1 study did so for the GAD-2, and no study reported evaluation metrics for the PHQ-2. A more accurate representation of the questionnaire metrics, therefore, can be found in Table 4, especially GAD-7, GAD-2, and PHQ-2. Although studies included as part of Table 4 focus on general adult populations rather than student ones, their variety and sample size make it a more realistic and reliable estimate.

Given this, the sensitivity for all 4 models was higher than the sensitivity achieved by each questionnaire in Table 4. The NPV of each model was slightly smaller. However, an expected consequence of adjusting the thresholds to increase sensitivity is a drop in specificity—as fewer false negatives are predicted, the number of false positives increases. This is reflected in the low specificity, PPV, and accuracy values. Absolute values for false positives can be seen in Tables S1, S3, S5, and S7 in Multimedia Appendix 1 in the upper right corner. The end goal of our system is not to conduct clinical triage but to provide students with a tool that recommends stepped care levels based on current needs. The ML models assess future risk to recommend lower-intensity, self-guided resources to students—which could mitigate risk through proactive prevention to guard against the development of symptoms and need for more intensive support. In this manner, having a higher number of false positives was deemed acceptable by clinician partners in the study, as this would not cause an undue burden on health care resources and might potentially reduce it. At worst, students who might not need any recommendation (false positives) would be recommended self-guided or nonclinical (eg, peer support) interventions. In other words, since these recommendations do not involve the use of clinical services, this would not increase the burden on already strained resources—in fact, if effective, the burden downstream on services would be expected to decrease with improved drinking behavior from the low-intensity interventions.

The features that contribute to the GAD-7 model also contribute to the GAD-2, albeit in a different order—for example, item 4 of the PSS-4 is the fifth most important contributor in the GAD-2 model but is in the 10th position on the GAD-7 model—suggesting that while these features are stable contributors to anxiety, interaction between features may also play a crucial role in determining their importance. As previously noted, the results of the GAD-2 and GAD-7 models were similar, indicating that other variables “compensate” for the removal of GAD-7 items. Indeed, for the GAD-2 model, the 2 most important features are similar to the previous model when removing items 3 and 5 of the GAD-7 as inputs. For example, the second most important feature of the GAD-7 model, lifetime anxiety history, became the most important feature of the GAD-2 model once item 3 of GAD-7 was not included as an input. Interestingly, the first item of GAD-7 (feeling nervous, anxious, or on edge) appears as the 13th most important feature, suggesting that the second GAD-7 item (not being able to stop or control worrying) is a much better predictor of future anxiety. The profile of the SHAP plot for PHQ-9 and PHQ-2 is different, with several features that did not strongly contribute to the PHQ-9 model appearing as the top contributors for the PHQ-2 model (such as sexual abuse and visit to emergency due to mental health problems). However, the PHQ-2 model still performed well, suggesting that the interaction between remaining features played a crucial role in predictions.

As outcomes vary, feature importance also changes. However, some similarities can be found when looking into the models grouped by depression or anxiety. Factors such as diagnosis of anxiety disorder, overwhelming stress as measured by PSS-4 items (in particular, items 3 and 4), and a family history of mood and anxiety disorders seem to be stable predictors of student anxiety levels. When predicting depression, PSS-4 items (in particular, item 4), history of childhood abuse, and emergency mental health visits also were stable predictors. These features could be collected by different sites trying to implement a similar survey and recommendation infrastructure.

It is important to note that to calculate the SHAP values in this paper, an average of all predictions was used, representing data from different users. In the future, if a platform was able to collect enough data from 1 student, it would be interesting to calculate SHAP values using an average of predictions from that specific student. This would provide a personalized ranking of the most important features for that student and, as such, would allow more personalized and tailored recommendations. As an example, in case a student is signposted to self-guided support, and in their personalized feature ranking item 2 of GAD-7 seems to be a major predictor, self-guided resources focusing on this item could be provided. On the same token, if a student indicated family history of a mental disorder, specific resources and services that deal with the issue can be recommended. This, however, would be dependent on having enough individual student data.

### Limitations and Future Work

Of note, this work relied on self-report data to develop rule-based algorithm and prediction models. Therefore, for future refinement and validation the prediction models will need to be compared with clinical assessment (ie, structured clinical interviews) and the concordance of the automated recommendations compared with consensus clinical judgment. Future work in the system should also focus on tier 2 levels (Figure 2), which take into account additional features that could further personalize treatment recommendations. For example, if a student is recommended for counseling and has a history of abuse, a counselor who specializes in this area could be highlighted. Future research should also investigate in more detail the longitudinal impact of mental health and the proposed intervention, that is, beyond the academic year.

Students with lived experience collaborated on the U-Flourish Survey study, defining and customizing the stepped care model, and drafting appropriate messaging as part of the U-Flourish research program. Future research on the recommendation engine focusing on refined development of accuracy and independent validation will continue to partner with student users, specifically, in refining the design, customization aspects (tier 2), and field testing. Furthermore, students from minoritized subgroups will continue to be involved in the creation of proper student-centric language for the recommendations to ensure culturally sensitive and engaging resources and that messaging is clear around automated suggestions based on shared, anonymized data. Since a conscious decision was made in this study to prioritize sensitivity over specificity, proper student-centric language is also essential to ensure no unnecessary stress to students who are classified at a potential future risk with the ML models.

When looking into the demographics (Table 5), the majority of respondents in the dataset were White and female for every group in the analysis, limiting generalizability. Therefore, we plan to test the algorithm and models on similar datasets collected from other universities and in different regions to ensure that they can be used accurately in populations with different characteristics. In particular, we are currently evaluating our system with a similar dataset to U-Flourish collected at the University of Oxford in United Kingdom [80]. Harmonized datasets will then be enriched with other data from subsets of students including clinical structured interviews, unstructured qualitative interviews, and linked medical records service use data. Once the application is further co-designed with students and validated with this larger harmonized and enriched datasets, we plan to carry out a rigorous multisite validation and implementation study in new cohorts of students at different institutions in different countries. Depending on privacy regulations, data could be stored locally at institutional servers as the infrastructure required to run these models would be minimal. Once implemented, we plan to monitor and investigate the longitudinal impact of the use of the application on early detection and prevention of anxiety and depression. Secondary outcomes will include effect on academic performance and well-being. Data collected as part of this deployment, properly anonymized, could also be used to improve prediction models and obtain a better balance between sensitivity and specificity and tailored recommendations.

Finally, it should be noted that while the prediction and recommendation engine models currently rely on self-report, the measures used are well validated and widely used in literature. We believe that their use is justified, given that the goal of the recommendation engine is to connect students to appropriate resources based on their symptoms without increasing the burden on health care services (ie, it is not intended as a diagnostic tool but as a screening measure to help connect high-risk students to services). That said, the measures could be further refined in terms of appropriate thresholds and the relative weighting of particular items in this target population. Furthermore, validation of the mental health calculator and recommendation engine is required in field studies using structured clinical interviews, which would further inform weighting and potential bias mitigation strategies. Field studies must also consider security and privacy risks. While outside the scope of this paper, a real-world implementation of the recommendation engine must account for secure data storage and collection. The predictive models should be housed in secure servers and receive only deidentified data as input to improve user safety. Any real-world deployment must also be constantly evaluated to identify and act on model drift (ie, changes in conditions that could lead to degradation in model performance) if it occurs.

### Conclusions

Based on data from the U-Flourish longitudinal successive cohort survey study, we were able to develop prediction models and a support recommendation engine that integrates pragmatic clinical rule-based algorithms and ML models to identify current and future risk of anxiety and depression and signpost students to the indicated level of mental health support, improving early detection and proactive prevention. Using a stepped care model and signposting to indicated level of support will thereby rationalize service use and improve support capacity. resources. Collectively, the U-Flourish mental health calculator (classification, prediction, and recommendation engine) is an accessible and sustainable digital solution addressing barriers to care and tailored for university students with common mental health concerns.

## Appendix

Multimedia Appendix 1 Machine learning models additional material.

## Abbreviations

C-SSRS: Columbia-Suicide Severity Rating Scale
GAD-2: 2-item Generalized Anxiety Disorder Questionnaire
GAD-7: 7-item Generalized Anxiety Disorder Questionnaire
ML: machine learning
NPV: negative predictive value
PHQ-2: 2-item Patient Health Questionnaire
PHQ-9: 9-item Patient Health Questionnaire
PPV: positive predictive value
PSS-4: Perceived Stress Scale–4
SHAP: Shapley additive explanations
