# Supplementary material for: Development of a Recommendation Engine to University Student Mental Health Support Aligned With Stepped Care: Longitudinal Cohort Study
Source: J Med Internet Res. 2025 Sep 17;27:e72669. doi: 10.2196/72669 (PMC12489415; doi:10.2196/72669)
Supplement: Multimedia Appendix 1 [file jmir_v27i1e72669_app1.docx]

## Multimedia Appendix 1: ML Models Additional Material

Table S1. Anxiety prediction—using GAD-7 inputs—contingency table.

|  | **# of Predictions for Class 0** | **# of Predictions for Class 1** |
| --- | --- | --- |
| **0** | 128 | 410 |
| **1** | 15 | 133 |

Table S2. Anxiety prediction—using GAD-7 inputs—evaluation metrics.

|  | **Precision** | **Recall** | **F1-score** | **Support** |
| --- | --- | --- | --- | --- |
| **0** | 0.90 | 0.24 | 0.38 | 538 |
| **1** | 0.24 | 0.90 | 0.38 | 148 |
| **Accuracy** |  | | 0.38 | 686 |
| **Macro Average** | 0.57 | 0.57 | 0.38 | 686 |


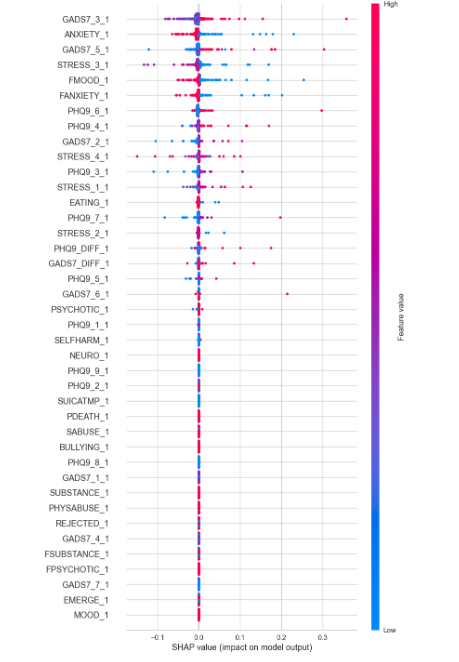


Figure S1. Anxiety prediction—using GAD-7 inputs—SHAP values.

Table S3. Anxiety prediction—using GAD-2 inputs—contingency table.

|  | **# of Predictions for Class 0** | **# of Predictions for Class 1** |
| --- | --- | --- |
| **0** | 103 | 435 |
| **1** | 12 | 136 |

Table S4. Anxiety prediction—using GAD-2 inputs—evaluation metrics.

|  | **Precision** | **Recall** | **F1-score** | **Support** |
| --- | --- | --- | --- | --- |
| **0** | 0.90 | 0.19 | 0.32 | 538 |
| **1** | 0.43 | 0.92 | 0.38 | 148 |
| **Accuracy** |  | | 0.35 | 686 |
| **Macro Average** | 0.57 | 0.56 | 0.35 | 686 |


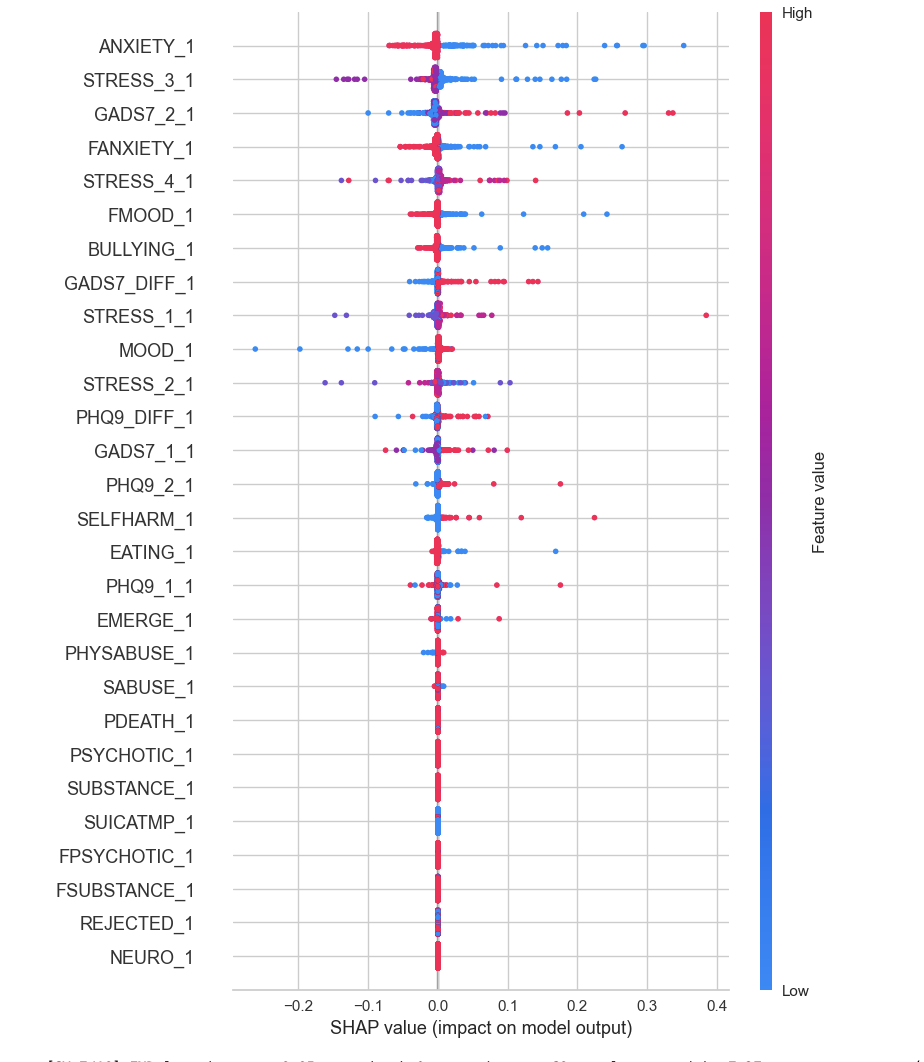


Figure S2. GAD-2 results—SHAP values.

Table S5. Depression prediction—using PHQ-9 inputs—contingency table.

|  | **# of Predictions for Class 0** | **# of Predictions for Class 1** |
| --- | --- | --- |
| **0** | 100 | 442 |
| **1** | 8 | 136 |

Table S6. Depression prediction—using PHQ-9 inputs—evaluation metrics.

|  | **Precision** | **Recall** | **F1-score** | **Support** |
| --- | --- | --- | --- | --- |
| **0** | 0.93 | 0.18 | 0.31 | 542 |
| **1** | 0.24 | 0.94 | 0.38 | 144 |
| **Accuracy** |  | | 0.34 | 686 |
| **Macro Average** | 0.58 | 0.56 | 0.34 | 686 |


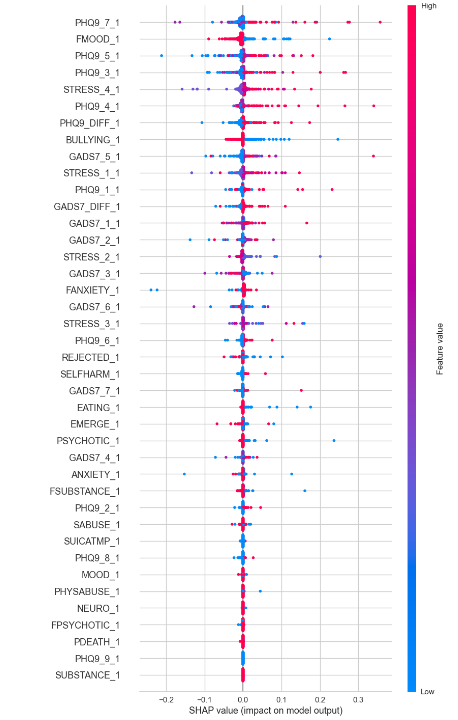


Figure S3. PHQ-9 results—SHAP values.

Table S7. Depression prediction—using PHQ-2 inputs—contingency table.

|  | **# of Predictions for Class 0** | **# of Predictions for Class 1** |
| --- | --- | --- |
| **0** | 108 | 434 |
| **1** | 14 | 130 |

**Threshold = 0.11**

Table S8. Depression prediction—using PHQ-2 inputs—evaluation metrics.

|  | **Precision** | **Recall** | **F1-score** | **Support** |
| --- | --- | --- | --- | --- |
| **0** | 0.89 | 0.20 | 0.33 | 542 |
| **1** | 0.23 | 0.90 | 0.37 | 144 |
| **Accuracy** |  | | 0.35 | 686 |
| **Macro Average** | 0.56 | 0.55 | 0.34 | 686 |


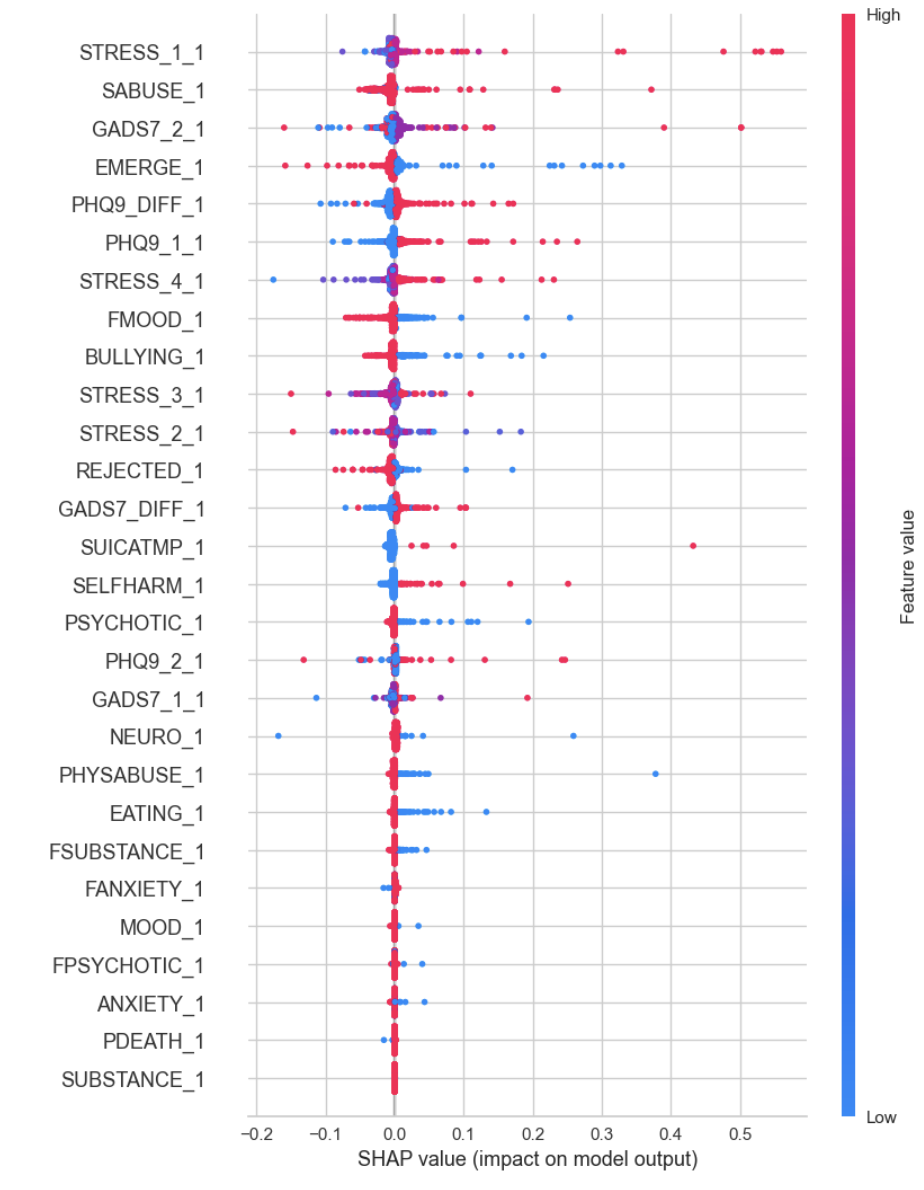


Figure S4. PHQ-2 results—SHAP values.
